# Cytogenetic anomalies are the predominant genetic alteration in children with nonfamilial tall stature: a comparative study with familial cases

**DOI:** 10.1007/s00431-025-06256-9

**Published:** 2025-06-17

**Authors:** Katerina Gregorova, Lukas Plachy, Petra Dusatkova, Klara Maratova, Julia Martinkova, Jana Drabova, Vit Neuman, Stanislava Kolouskova, Marta Snajderova, Barbora Obermannova, Jan Lebl, Zdenek Sumnik, Stepanka Pruhova

**Affiliations:** 1 Department of Pediatrics, Second Faculty of Medicine, Charles University and University Hospital Motol, V Uvalu 84, Prague 5, 150 06 Czech Republic; 2 Department of Biology and Medical Genetics, Second Faculty of Medicine, Charles University and University Hospital Motol, V Uvalu 84, Prague 5, 150 06 Czech Republic

**Keywords:** Non-familial tall stature, Tall stature, Growth, Genetics of tall stature, Next-generation sequencing

## Abstract

The purpose of this study is to elucidate the genetic causes and phenotypic presentation of nonfamilial tall stature (nFTS) and to compare these findings with those of familial tall stature (FTS) from the same population that was previously studied. Children with nFTS (defined as a height >  + 2 SDs with both parents’ heights <  + 2 SDs) underwent endocrine and anthropometric examinations and genetic testing (karyotyping, *SHOX* gene dosage analysis and next-generation sequencing of 786 growth-associated genes). Exome sequencing was performed in patients with negative genetic results and a height >  + 3 SDs. A total of 55 children with nFTS were enrolled. The median height was + 2.8 SD (2.4–3.2 SD), and the median midparental height was + 0.7 SD (0.4–0.9 SD). Genetic causes of tall stature were identified in 6/55 (11%) children. Specifically, four children had gonosomal aneuploidy (47,XXY [2x], 47,XXX, 48,XXXX), one had a heterozygous complex rearrangement including *SHOX* gene duplication, and one carried a pathogenic variant in the *TGFBR2* gene leading to Loeys–Dietz syndrome. A genetic cause of tall stature was significantly less common in nFTS (11%) than in our previously published cohort with FTS (32%).

*Conclusion*: Cytogenetic abnormalities were the predominant genetic alteration identified in children with nFTS, confirming the justification of karyotype analysis in this cohort. The probability of genetic alterations was greater in children with FTS than in those with nFTS. Our findings suggest that the current guidelines for complex investigation are efficient for children with nFTS but need revision in children with FTS.

**Table:** 

**What is known – what is new** • *Although tall stature is generally considered beneficial, it can be associated with health risks which need to be recognized in time. Tall stature without intellectual impairment is usually considered to be polygenic*. • *However, the cause of familial tall stature was monogenic more often than it was thought previously*. • *Children with non-familial and apparently non-syndromic tall stature have never been systematically investigated*. • *Monogenic causes of non-familial tall stature were observed in 11% of patients, including a participant with Loeys–Dietz syndrome*.

**Supplementary Information:**

The online version contains supplementary material available at 10.1007/s00431-025-06256-9.

## Introduction

Tall stature (TS) is defined as growth >  + 2 standard deviations (SD) above the mean for age and sex [1–3]. As TS is frequently considered beneficial by society, it is not given as much attention as short stature is [4]. Children with TS are therefore usually not referred for endocrine testing. This approach is in accordance with current guidelines, which focus mainly on dysmorphic features and target height (TH) calculated from the parents’ heights. According to these guidelines, only tall children who have dysmorphic features or other abnormal findings and/or grow above the TH should be referred to a pediatric endocrinologist, and only then is a genetic examination considered [5–7]. However, as shown in our previous publication [8], this approach may lead to an underdiagnosis of the genetic causes of familial TS (FTS) associated with severe long-term complications.

Health risks related to TS may be endocrine in origin (e.g., precocious puberty, hyperthyroidism, growth hormone (GH) excess), musculoskeletal (scoliosis, joint diseases), oncological (e.g., severe risk of malignancy of overgrowth syndromes such as Sotos syndrome [9] or increasing risk of development of several types of cancer with each 10 cm above average height [10]), or cardiovascular (e.g., aortic aneurysm or valve dysfunction in people with connective tissue disorders such as Marfan syndrome [11] or Loeys–Dietz syndrome [12, 13]). Moreover, subjects with TSs generally have shorter lifespans than their peers with shorter heights do [14].

Genetic studies have demonstrated a potential to improve our knowledge regarding the etiology of TS. To date, the causes of TS have been studied mainly in patients with apparent syndromic features [15, 16]. Our previous work revealed that although most children with FTS (defined as a height >  + 2 SDs with at least one parent’s height >  + 2 SD) presented no or only subtle dysmorphic features, a genetic cause of their TS was determined in 11/34 (32%) of them [8]. Importantly, to the best of our knowledge, studies focusing on children with nonfamilial TS (nFTS) who do not exhibit profound dysmorphic features are lacking.

The aim of this study was to elucidate the genetic causes in children with nFTS, to describe their phenotype in detail, and to compare the results of nFTS cohorts to those of the FTS cohort from a previous study [8].

## Materials and methods

### Study settings and inclusion criteria

**Fig. 1:**
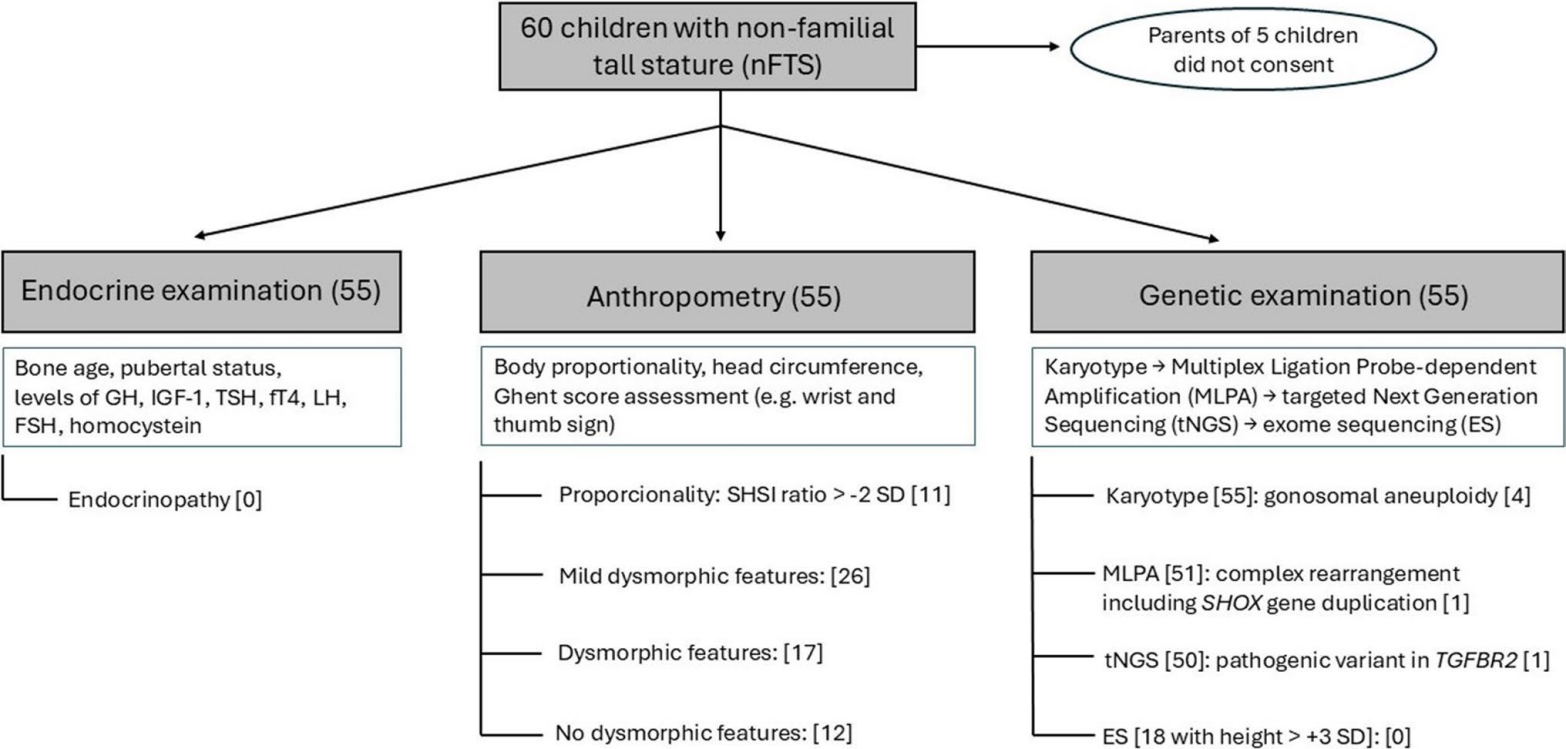
Flowchart of the investigation of patients with nFTS

This is a monocentric study conducted at Motol University Hospital, a tertiary clinical center in Prague, Czech Republic. The inclusion criteria for the nFTS study were as follows: children whose height was >  + 2 SDs and whose parents’ heights were both <  + 2 SD. These children were newly referred by general practitioners for TS or were already followed up in our outpatient clinic between September 2020 and April 2024. All children who fulfilled the criteria and whose parents or legal guardians signed written informed consent were enrolled in the study. The detailed flowchart of the study is shown in Fig. 1. The study was conducted in accordance with the Declaration of Helsinki and was approved by the Ethical Committee of Motol University Hospital, Czech Republic, issued on 17 June 2020. Data of probands investigated in this current study were subsequently compared to previous results of our study focused on children from the same population displaying FTS [8]. The inclusion criteria of children with FTS were as follows: (1) children followed up at our clinic having (2) life-maximum height > 2 SD for age, sex, and Czech population in both the child and his/her taller parent and (3) whose parents or legal guardians signed written informed consent with the study.

### Clinical examinations

Endocrine, anthropometric, and genetic investigations (except for exome sequencing [ES]—please see below) of children in the nFTS cohort were performed using the protocol reported previously for FTS patients [8].

All study participants were examined by an experienced pediatric endocrinologist. A detailed medical history was obtained from medical records and reported by the parents. The physical examination focused on pubertal development and apparent dysmorphic features. Biochemical examination was performed mainly to exclude hyperthyroidism and GH excess. A prolonged oral glucose tolerance test was performed on all children whose IGF-1 concentration was >  + 2 SDs for age and sex and whose basal GH concentration was > 1 µg/L [17]. Bone age was evaluated using the Tanner-Whitehouse method [18]. Consequently, all study participants were examined by a skilled clinical anthropologist trained in the recognition of syndromic clinical signs. The dysmorphic features were assessed using a scoring system evaluating the severity of craniofacial, orofacial, skeletal, skin, eye, and other signs (Table 1). In case of obtaining 1 to 3 points, the proband was described with mild dysmorphic features, while in the presence of 4 or more points, the proband was considered having dysmorphic features. All data were standardized according to the normative values [19–23]*.* The sitting height to subischial length (SHSI) ratio was not available for two subjects (no. 37 and 42) and birth parameters were not known in one patient (no. 39). Other participants had no missing growth, clinical, biochemical, and anthropometric data; therefore, the probability of influence of results because of missing data was low.

**Table 1:** Scoring system of dysmorphic features

Phenotypic features	Number of points: 0	Number of points: 1	Number of points: 2
Craniofacial	None	Mild anomalies (e.g., broad nasal bridge)	Marked features (e.g., dolichocephaly, macrocephaly, hypertelorism, frontal bossing)
Orofacial	None	Mild (e.g., high-arched palate)	Major dysmorphism (e.g., cleft palate, orofacial stigmatization)
Skeletal	None	Mild scoliosis, genua valga, clinodactyly, pedes calcaneovalgi	Pectus deformity, arachnodactyly, severe scoliosis
Skin/Connective tissue	None	Single feature (e.g., striae, mild hyperelasticity)	> 2 features (e.g., striae + joint hypermobility/skin hyperlaxity)
Eye	None	Myopia < 3D, astigmatism, amblyopia, strabism	Myopia > 3D, ptosis, epicanthus, lens dislocation, > 2 minor findings
Other	None	Isolated finding (e.g., disproportionality, hallux, 1 café au lait spot, fetal finger pads)	multiple minor findings (e.g., > 1 café au lait spots), hypotonia, macrosomia, gynoid habitus

Total score calculation and classification: 0 points → no dysmorphic features; 1–3 points → mild dysmorphic features; ≥ 4 points → dysmorphic features

### Genetic examination

All study participants first underwent standard karyotyping. Those with a normal karyotype were subsequently tested by Multiplex Ligation Probe-dependent Amplification (MLPA) for *SHOX* gene duplications or deletions (kit P018, MRC Holland, Amsterdam, Netherlands). The DNA of all the probands with a normal karyotype and the absence of *SHOX* gene duplication or deletion was subsequently analyzed using a custom targeted next-generation sequencing (NGS) panel (Agilent Technologies, Santa Clara, USA) of 786 genes associated with TS or involved in the development and regulation of the pituitary and growth plates [8]. ES was performed in 18 patients with height >  + 3 SDs and with no causal genetic variant detected. The threshold of >  + 3 SD was selected based on literature search that defined height above 3 SD as “extreme” or “severe” warranting further diagnostic evaluation and genetic testing [24]. ES was executed using the SureSelect Human All Exon Kit V6 + UTRs (Agilent Technologies, Santa Clara, USA). The indexed products of both the NGS panel and ES were sequenced by synthesis using NextSeq 550 or NextSeq 2000 genetic analyzers (Illumina, San Diego, USA) with 100X average coverage.

### Bioinformatic evaluation

Our bioinformatics pipeline for generating the NGS data was described previously [8]. Segregation of a single-nucleotide variant was confirmed in available parents using Sanger sequencing [25]. The American College of Medical Genetics and Genomics (ACMG) standards and guidelines [26, 27] were used for the final evaluation and classification of the detected variants into five groups: pathogenic, likely pathogenic, benign, likely benign, or variants of uncertain significance (VUS).

### Statistical analysis

Descriptive data were summarized as standard deviations related to the normative values with interquartile range for continuous variables and as absolute and relative frequencies in the case of categorical variables. Since the data were not normally distributed (as shown using histogram), statistical comparisons of the results from the nFTS and FTS cohorts were performed using the Mann–Whitney test (continuous variables). The chi-square test of independence was used for assessment of categorical variables. *P* values < 0.05 were considered statistically significant.

## Results

Of the 60 children who were recruited from our endocrine outpatient clinic for nFTS, 55 consented to participate in the study. The median age of the participants was 13 years (interquartile range 10–16 years), their body height was 2.8 SD (2.4–3.2 SD), their SHSI ratio was − 1.3 SD (− 2.0–0.8 SD), their birth length was 0.3 SD (− 0.1–1.0 SD), their birth weight was 0.8 SD (0.1–1.5 SD), and their midparental height (MPH) was 0.7 SD (0.4–0.9 SD). Dysmorphic signs were present in 43/55 (78%) of the children, of whom 26/43 (60%) were mild; therefore, they were revealed only by detailed anthropometric examination (Table 1). A significant medical history was present in five children who were followed up for cardiovascular conditions (foramen ovale apertum, essential hypertension, hemodynamically insignificant defect of the atrial septum, hypertrophy of the atrial septum, tricuspid insufficiency), and three had a history of tumors (low-grade glioma [2x], Burkitt lymphoma). No child was diagnosed with mental retardation or reported significant learning difficulties. Three children reported precocious puberty. However, as their TS persisted after long-term adequate treatment, another cause of TS was not excluded, and these children were therefore also included in the study.

A genetic cause of TS was identified in 6/55 (11%) children with nFTS. The majority (4/6, 67%) of the genetic alterations were discovered from karyotype investigations (karyotypes 47,XXY [2x], 47,XXX, 48,XXXX]). One genetic cause was found using MLPA (complex rearrangement including *SHOX* gene duplication), and one was found via an NGS panel (heterozygous pathogenic variant in the *TGFBR2* gene causing Loeys–Dietz syndrome)*.* Among the six children whose genetic etiology of TS was elucidated, four presented apparent syndromic features (probands no. 4, 6, 9, and 13) while two presented mild dysmorphic signs (probands no. 33 and 39). No (likely) pathogenic genetic aberrations associated with TS were discovered in children with positive cardiovascular or oncological history. VUS according to ACMG guidelines were identified in an additional 11/55 (20%) probands using an NGS panel and ES (encoding the genes *BMP4*,* FBN1*,* ZBTB20*,* MATN3*,* TET3*,* COL1A1*,* COL1A2*,* COL2A1*,* COL6A3*,* COL11A1*, and *ADAMTSL4*; Supplementary Table 1).

A comparison of the current results of patients with nFTS and our previously published FTS cohort [8] revealed that children with nFTS were born with significantly shorter birth lengths and achieved lower maximal heights (Table 2). A genetic cause of TS was discovered less frequently in children with nFTS (11%) than in probands with FTS (32%). Variants causing FTS that also increase the risk of oncologic disorders were found in the *NSD1* and *GPC3* genes, respectively, in the cohort of patients with FTS. However, no (likely) pathogenic variants, nor VUS, were observed in the respective genes in the nFTS cohort using the same methodology. Detailed comparisons of the clinical and genetic data from the nFTS and FTS cohorts are summarized in Table 2.

**Table 2:** Comparison of clinical and genetic findings in FTS and nfTS cohorts

	FTS (*n* = 34)	nfTS (*n* = 55)	***P*-**value
Sex (female/male)	19/15	31/24	0.863
Age (years)*	13.6 (11.0–16.8)	13.0 (10.0–16.0)	0.294
Birth length (SD)*	0.8 (0.5–1.6)	0.3 (− 0.1–1.0)	**0.007**
Birth weight (SD)*	1.1 (0.4–0.8)	0.8 (0.1–1.5)	0.682
Maximal height (SD)*	3.1 (2.7–3.6)	2.8 (2.4–3.2)	**0.006**
Sitting height to subischial length (SD)*	− 1.7 (− 2.2–0.7)	− 1.3 (− 2.0–0.8)	0.267
(Mild) dysmorphology (%)	70.6	78.2	0.578
IGF-1 level (SD)*	0.7 (− 0.03–1.5)	0.8 (0.2–1.9)	0.689
Mid-parental height (SD)*	1.9 (1.6–2.3)	0.7 (0.4–0.9)	** < 0.001**
Detected genetic cause of TS (%)	32.4	10.9	**0.026**
Gonosomal aneuploidy (%)	5.9	7.3	0.863
Complex rearrangement including *SHOX* gene duplication (%)	2.9	1.8	0.680
Monogenic (likely) pathogenic variant (%)	23.5	1.8	**0.003**
Genetic syndrome with high oncologic risks (%)	8.8	0	NA
VUS in genes associated with connective tissue disorders (%)	11.8	12.7	0.933

*Data displayed as SD with interquartile range

## Discussion

In the present study, the genetic etiology of TS was elucidated in 11% of the children with nFTS. To the best of our knowledge, no other study has focused exclusively on the genetic causes of TS in persons without a family history of TS. However, a study by Albuquerque et al. [16] identified a subgroup of five patients with non-syndromic nFTS among 12 subjects with non-syndromic TS. The genetic cause was revealed in only one patient (1/12; 8.3%) from the respective non-syndromic cohort with TS (47,XYY), although his parents’ heights were not known [16].

Understanding the etiology of TS is important because of possible related clinical conditions. Gonosomal aneuploidies detected in 67% of the probands with observed genetic causes of nFTS could be associated with behavior disorders and infertility [28, 29]. Therefore, a karyotype investigation is justified in children with nFTS. One child with nFTS was confirmed to have Loeys–Dietz syndrome, which is known to be associated with severe cardiovascular complications [12].

Although the presence of mild dysmorphic signs was frequently documented among children with nFTS (Supplementary Table 1), these signs demonstrated only a limited capacity to predict positive genetic test results. For example, dysmorphic signs suggestive of connective tissue disorders were observed in 53% (29/55) of the probands. However, connective tissue disorders were identified genetically in only one patient (with Loyes–Dietz syndrome). Notably, of the 29 children with nFTS with dysmorphic features resembling connective tissue disorders, 7 had VUSs of genes known to be related to connective tissue (*BMP4*,* MATN3*,* COL2A1*,* COL11A1*,* COL1A1*,* COL1A2*, and *COL6A3*). Although the pathogenicity of several variants remained uncertain according to current ACMG guidelines, we believe that some of the respective alterations may represent variants with phenotypic relevance suggesting the carriers warrant close monitoring, family segregation analysis, and possibly obtain future variant reclassification as more evidence accumulates. For example, patient no. 11 with a VUS in the *COL2A1* gene exhibited multiple marfanoid features (arachnodactyly, striae, joint hypermobility, skin laxity, high-arched palate), including a Ghent score of 7, suggesting a clinically significant connective tissue disorder, which was previously described in TS and marfanoid habitus by Demal et al. [30]. In addition, the observed variant c.133G > T in the *COL2A1* gene was reported in population variant database in the low representation (gnomAD v4.1.0 (European, non-Finnish): 0.00025%); the missense variants feature a common mechanism of gene disruption and several in silico prediction tools (DANN, SIFT, PolyPhen-2) predicted deleterious effect on protein. On the other hand, all in silico predictions were not uniform (Revel). The variant was found in the mother of the patient with SD height + 1.74. Unavailable detailed clinical investigation of her currently does not allow a clear conclusion regarding segregation. The respective variant was reported in the ClinVar database as likely benign however by only single user without additional clinical data. Another such example can be seen in patient no. 17 who carried a VUS in *COL1A1*, a gene associated with Ehlers–Danlos syndrome and osteogenesis imperfecta, who presented with striae and positive wrist sign. The pathogenicity of variant c.2420C > A in the *COL1A1* gene speaks its absence in the variant population databases (gnomAD v4.1.0), damaging in silico predictions by majority of tools (Revel, CADD, DANN, SIFT, PolyPhen-2), type of variant (missense), and possible location in an important domain. These facts are however currently not sufficient to classify the variant as likely pathogenic also because the variant is carried by the participant’s mother with height SD + 1.55. We consider this variant as a “hot VUS” that has the highest chance in our dataset to be reclassified as likely pathogenic in the future. The connection to TS can be link to the fact that increased procollagen 1A1 was found in the fibroblasts of patients with TS in a study by Gupta et al. [31]. The disruption of the *COL11A1* gene is associated with Marshall-Stickler syndrome which could be of a potential interest given the observed joint hypermobility and skin hyperlaxity in patient no. 43 with VUS c.1351-3 T > A. This variant has been three times classified as VUS in the ClinVar database and was observed in three subjects in the gnomAD database v4.1.0 (0.0003%; European, non-Finnish). In silico tools predict altered splicing (SpliceAI). Patient no. 2 also displayed a phenotype of marfanoid features resembling a connective tissue disorder. She carried a VUS in the *ADAMTSL4* gene known to cause isolated ectopia lentis by interacting with microfibrils of fibrillin 1 [32]. Although this interaction has been shown only in the eye [33], it might affect the formation of other fibrillin molecules throughout the body. The heterozygous missense change c.2398G > A in the *ADAMTSL4* gene is suggested to be disruptive by many in silico prediction tools (Revel, DANN, SIFT, PolyPhen-2, Align-GVGD); however, it was observed in population variant database (gnomAD v4.1.0 (European, non-Finnish): 0.016%). Since ADAMTSL4-related conditions display autosomal recessive mode of inheritance (OMIM # 610,113), we might speculate that second heterozygous variant might be hidden, for example, in the deep intronic or regulatory regions of the gene.

The overall detection rate of genetically caused TS was significantly lower in the nFTS cohort (11%) than in patients from the same population with FTS (32%) [8]. A point pathogenic variant was observed in only one out of 55 patients (2%) with nFTS compared with eight out of 34 patients (24%) with FTS using the same NGS panel. Reasons for a lower detection rate of single gene variants in patients with nFTS might include polygenic traits of inheritance [34], unobserved noncoding variants, or hidden monogenic causes of TS in genes that have not yet been fully associated with TS and are currently classified as VUS. Apart from obvious clinical differences between patients with nFTS and FTS, such as parental height, children with nFTS had a smaller median height and disproportionately lower body (with shorter lower limbs) than did those with FTS (Table 2), indicating that children with FTS were referred to specialists more often with more severe clinical characteristics than were children with nFTS. These data suggest that the current guidelines [5–7] for the comprehensive examination of nFTS, which recommend genetic testing based on dysmorphic features or growth above TH, are adequate. Following these guidelines would also lead to revealing the chromosomal aneuploidies of probands from our study, mainly due to the presence of dysmorphic features. On the other hand, a comparison of our two studies also revealed that these guidelines are not efficient in the case of FTS, as these children usually grow in accordance with their TH, and their dysmorphic signs can be underestimated because of the presence of similar signs in their parents. Taken together, the results of this study and our previous study suggest that more attention should be given to the group of children with FTS, which are paradoxically often overlooked in detailed examinations because parent(s) with TS have healthy children with TS.

Based on results of our studies, pediatricians should consider referring children with nFTS even in the absence of obvious syndromic features to pediatric endocrinologists and/or clinical geneticists to indicate karyotype examination because of possible gonosomal aneuploidy. More attention should be given to children with FTS, especially when mild dysmorphic features or disproportional growth are present, as monogenic causes are more common in these families than previously assumed. Therefore, referral to a pediatric endocrinologist, clinical anthropologist, and clinical geneticist is advisable. In both groups, pediatricians should not rely solely on apparent normal psychosocial development or “benign” family history to rule out underlying pathology. This stepwise approach may help to identify hidden conditions with long-term health consequences and improve individualized care in children with TS.

We acknowledge that our study has several limitations. First, our detailed anthropometric examination was only performed on the children but not on the children’s parents. Additionally, the genetic methods used did not cover all possible genetic causes of disorders, such as noncoding variants (except for disruptions in the exon‒intron boundaries up to a distance of 10 nucleotides), variants in uncovered regions using selected methods, variants in the length of repetitive regions, and low-level chromosome mosaicism. Moreover, only a limited number of children with nFTS from a single center were examined in our study, and no matched or randomized control group was investigated. Furthermore, the comparison of children with nFTS to a previously studied cohort with FTS was not a prospectively matched or randomized. Therefore, potential selection bias or baseline differences between groups must be considered when interpreting the comparative results. The absence of trio exome sequencing because of the unavailability of the DNA samples of some of the parents might influence the interpretation of variants. Finally, the Czech population is very homogenous, belonging to the European, non-Finnish genetic ancestry group, which may limit the generalizability of the results. Our results, therefore, need to be confirmed by larger studies.

## Conclusion

A single genetic cause of TS with additional possible clinical implications was revealed in 6/55 (11%) children with nFTS. The probability of finding a genetic cause of TS was significantly lower in children with nFTS than in those in our previous study with children with FTS.

## Supplementary Information

Below is the link to the electronic supplementary material.

Supplementary1 (PDF 451 KB)

## Data Availability

The data that support the findings of this study are not openly available due to reasons of sensitivity and are available from the corresponding author upon request.

## Abbreviations

ACMG: American College of Medical Genetics
ES: Exome sequencing
FTS: Familial tall stature
GH: Growth hormone
MLPA: Multiplex ligation probe-dependent amplification
nFTS: Nonfamilial tall stature
NGS: Next-generation sequencing
SHSI: Sitting height-to-subischial length ratio
SD: Standard deviation
TH: Targeted height
TS: Tall stature
VUS: Variant of uncertain significance
