# Supplementary material for: Cytogenetic anomalies are the predominant genetic alteration in children with nonfamilial tall stature: a comparative study with familial cases
Source: Eur J Pediatr. 2025 Jun 17;184(7):423. doi: 10.1007/s00431-025-06256-9 (PMC12170751; doi:10.1007/s00431-025-06256-9)
Supplement: Supplementary file 1 — Supplementary1 (PDF 451 KB) [file 431_2025_6256_MOESM1_ESM.pdf]

**Cytogenetic anomalies are the predominant genetic alteration in children with nonfamilial tall stature: a comparative study with familial cases**

Katerina Gregorova, Lukas Plachy, Petra Dusatkova, Klara Maratova, Julia Martinkova, Jana Drabova, Vit Neuman, Stanislava Kolouskova, Marta Snajderova, Barbora Obermannova, Jan Lebl, Zdenek Sumnik, Stepanka Pruhova

**Supplementary Table 1. Medical history, phenotypic features, and genetic results of participants**

Abbreviations: ACMG: American College of Medical Genetics and Genomics (ACMG) standards and guidelines; AD: autosomal dominant inheritance; ADHD: attention deficit hyperactivity disorder; AITD: autoimmune thyroid disease; AR: autosomal recessive inheritance; D: diopter; F: female; FoA: foramen ovale appertum; het: heterozygous; LP: likely pathogenic; M: male; Ma: maternal; MPH: midparental height; NA: not available; P: pathogenic; Pa: paternal; PD: pseudoautosomal dominant; SD: standard deviation; SHSI: Sitting height to subischial length ratio; T1D: diabetes mellitus type 1, VUS: variant of uncertain significance

| Patient number | Gender | Age (years) | Height (SD) | Mother's height (SD) | Father's height (SD) | MPH (SD) | IGF-1 (SD) | Birth weight (SD) | Birth length (SD) | SHSI (SD) | Significant medical history | Dysmorphic features | Suggestive diagnosis | Gene | Associated syndrome | Transcript of the genes | Nucleotide level * | Protein level | ACMG classification | Variant zygosity | Mode of inheritance of the syndrome | Segregation in parents |
|---|---|---|---|---|---|---|---|---|---|---|---|---|---|---|---|---|---|---|---|---|---|---|
| **Children with dysmorphic features** | | | | | | | | | | | | | | | | | | | | | | |
| **1** | F | 7 | 2.1 | 1.1 | 0.7 | 0.9 | 0.3 | 0.4 | -0.4 | -2.0 | - | Pectus excavatum, astigmatism | Connective tissue disorder: Marfanoid habitus | - | - | - | - | - | - | - | - | - |
| **2** | F | 13 | 4.5 | 1.6 | 1.8 | 1.8 | 3.3 | 2.1 | 1.4 | -1.3 | Low grade glioma | Striae, gothic palate, positive thumb and wrist sign, genua valga | Connective tissue disorder: Marfanoid habitus | *ADAMTSL4* | Ectopia lentis | NM_019032.6 | c.2398G>A | p.Gly800Ser | VUS | het | AR | - |
| **3** | F | 14 | 2.4 | 0.9 | 0.2 | 0.6 | -0.7 | 2.3 | 1.9 | -1.0 | - | Severe scoliosis, positive thumb and wrist sign, joint hypermobility, Ghent´s criteria 5 points | Connective tissue disorder: Marfanoid habitus | - | - | - | - | - | - | - | - | - |
| **4** | F | 16 | 3.2 | 1.9 | 1.7 | 1.9 | 0.4 | -0.3 | 0.5 | 0.6 | - | Striae, hallux, orofacial stigmatization | - | *SHOX* | - | NM_000451.3 | rsa[GRCh38] Xp22.33(307456_751604x3,781201_850607x4,899343x3,963712x2) | - | LP | het | PD | - |
| **5** | M | 17 | 2.7 | 0.4 | 0.7 | 0.6 | -1.4 | 1.9 | 0.7 | -1.3 | Aortic root dilatation, aortal and mitral regurgitation | Chest asymmetry, striae, positive thumb and wrist sign, high- arched palate | Connective tissue disorder: Marfanoid habitus | *FBN1* | Marfan syndrome | NM_000138.5 | c.7819+6del | p.? | VUS | het | AD | De novo |
| **6** | F | 5 | 2.5 | 0.9 | -0.6 | 0.1 | 0.2 | -0.6 | -1.3 | 0.4 | Obesity | Lower set ears, down slanting palpebral fissures, macrosomia, thin lips | - | 47,XXX | Trisomy X syndrome | - | - | - | P | - | - | - |

| | | | | | | | | | | | | | | | | | | | | | | |
|---|---|---|---|---|---|---|---|---|---|---|---|---|---|---|---|---|---|---|---|---|---|---|
| **7** | M | 15 | 2.3 | 0.4 | 1.0 | 0.7 | 0.9 | 0.8 | 1.6 | -0.4 | - | Striae, mild scoliosis, macrocephaly | - | *MATN3* | Multiple Epiphyseal dysplasia type 5 | NM_002381.5 | c.541C>T | p.Arg181Ter | VUS | het | AD | Ma |
| **8** | M | 14 | 2.8 | 0.1 | 1.8 | 1.0 | 0.4 | 2.7 | 1.1 | -2.0 | Myopia (-0.8 D) | Chest deformity, striae, mild scoliosis, positive thumb and wrist sign, joint hypermobility, skin hyper elasticity | Connective tissue disorder: Marfanoid habitus | - | - | - | - | - | - | - | - | - |
| **9** | F | 16 | 2.8 | 1.8 | 0.7 | 1.3 | -0.7 | 0.2 | -0.4 | -2.1 | - | Pedes calcaneovalgi, brachydactyly, hypotonia, joint hypermobility | Connective tissue disorder | 48,XXXX | Tetrasomy X syndrome | - | - | - | P | - | - | - |
| **10** | M | 16 | 3.7 | 1.7 | 1.1 | 1.3 | 1.1 | 0.1 | 0.2 | 2.6 | Mitral regurgitation | Positive wrist sign, striae, gynoid habitus | Klinefelter syndrome phenotype | - | - | - | - | - | - | - | - | - |
| **11** | F | 15 | 2.4 | 1.7 | -0.2 | 0.8 | 0.8 | -0.1 | 0.5 | -1.5 | - | Dolichocephaly, arachnodactyly, joint hypermobility, striae, skin hyper elasticity, high-arched palate, Ghent score 7 points | Connective tissue disorder: Marfanoid habitus | *COL2A1* | Stickler syndrome | NM_001844.5 | c.133G>T | p.Asp45Tyr | VUS | het | AD | Ma |
| **12** | F | 13 | 2.4 | 1.2 | -1.0 | 0.04 | 1.9 | 0.03 | -0.4 | -1.4 | - | Striae, joint hypermobility, mild facial stigmatization, dolichocephaly, protruding acorns | Connective tissue disorder | - | - | - | - | - | - | - | - | - |
| **13** | M | 15 | 2.5 | 1.7 | NA | NA | 0.6 | -0.6 | 2.1 | -2.8 | - | Lumbar hyperlordosis, striae, joint hyperlaxity in elbows, genua valga | Connective tissue disorder | 47,XXY | Klinefelter syndrome | - | - | - | P | - | - | - |

| | | | | | | | | | | | | | | | | | | | | | | |
|---|---|---|---|---|---|---|---|---|---|---|---|---|---|---|---|---|---|---|---|---|---|---|
| **14** | F | 16 | 2.2 | -0.04 | 0.2 | 0.1 | 0.7 | 0.8 | 0.1 | -2.1 | myopia (-1D/-1D) | Striae, positive thumb and wrist sign, joint hypermobility | Connective tissue disorder | - | - | - | - | - | - | - | - | - |
| **15** | F | 10 | 2.2 | 0.1 | 1.5 | 0.9 | 2 | 2.1 | 0.5 | -0.6 | Macrosomia | Joint hypermobility, high-arched palate, broad nasal root | Connective tissue disorder | - | - | - | - | - | - | - | - | - |
| **16** | M | 13 | 3.4 | 1.4 | 0.5 | 0.9 | 1.5 | 0.7 | 1.1 | -2.0 | - | Striae, skin hyper elasticity, joint hypermobility, positive thumb sign, pectus carinatum | Connective tissue disorder | - | - | - | - | - | - | - | - | - |
| **17** | F | 15 | 3.2 | 1.6 | 1.3 | 1.5 | 1.3 | 1.4 | 1.0 | -2.1 | Myopia (-1.3/-1.5 D) | Striae, positive wrist sign | Connective tissue disorder | *COL1A1* | Ehlers-Danlos syndrome, Osteogenesis imperfecta type 1,2,3, 4 | NM_000088.4 | c.2420C>A | p.Pro807His | VUS | het | AD | Ma |
| | | **Children with mild dysmorphic features** | | | | | | | | | | | | | | | | | | | | |
| **18** | F | 15 | 2.9 | 0.8 | 0.4 | 0.6 | 0.9 | 1.5 | 0.5 | -1.4 | | Positive thumb and wrist sign, striae, clinodactyly | Connective tissue disorder: Marfanoid habitus | - | - | - | - | - | - | - | - | - |
| **19** | M | 19 | 3.2 | 0.9 | -1.0 | -0.1 | -1.5 | 1.7 | 0.2 | -0.7 | Low grade glioma in optic chiasm | Striae, borderline positivity of wrist sign | - | - | - | - | - | - | - | - | - | - |
| **20** | M | 19 | 2.4 | 0.6 | 0.4 | 0.5 | 2.7 | -0.3 | -0.2 | -0.8 | T1D | Higher elasticity of skin, striae, positive wrist sign | Connective tissue disorder | - | - | - | - | - | - | - | - | - |
| **21** | M | 14 | 2.8 | -0.7 | 0.8 | 0.1 | 1.2 | 0.6 | 2.1 | -0.2 | - | Skin hyperelasticity, borderline wrist and thumb sign, joint hypermobility | Connective tissue disorder | - | - | - | - | - | - | - | - | - |

| | | | | | | | | | | | | | | | | | | | | | | |
|---|---|---|---|---|---|---|---|---|---|---|---|---|---|---|---|---|---|---|---|---|---|---|
| **22** | M | 9 | 2.1 | -0.04 | 0.7 | 0.3 | -0.3 | 0.5 | 0.7 | -1.2 | FoA | Fetal finger pads | - | - | - | - | - | - | - | - | - | - |
| **23** | F | 20 | 2.1 | 0.6 | 0.4 | 0.5 | -0.5 | 1.1 | 0.1 | 0.5 | Sklerodermia, mental anorexia, myopia (-1D) | Striae | - | - | - | - | - | - | - | - | - | - |
| **24** | M | 18 | 3 | 0.4 | 0.7 | 0.5 | -0.6 | -1.0 | -0.7 | -1.3 | Undescended testicles | Striae, mild scoliosis | - | - | - | - | - | - | - | - | - | - |
| **25** | F | 10 | 2.1 | 0.1 | 0.7 | 0.4 | 2.4 | 1.9 | 1.0 | -1.1 | - | Joint hypermobility, positive thumb and wrist sign | Connective tissue disorder | - | - | - | - | - | - | - | - | - |
| **26** | M | 14 | 3.6 | 0.6 | 1.1 | 0.8 | -1.1 | 1.3 | 0.7 | -1.3 | Obesity, precocious puberty | Striae, high-arched palate | Connective tissue disorder | *BMP4* | - | NM_001202.6 | c.1121T>C | p.Val374Ala | VUS | het | - | - |
| **27** | M | 18 | 4.1 | 1.7 | 1.4 | 1.5 | 2 | 0.6 | 0.2 | -2.5 | Left testicular agenesis,ventricular septal defect, myopia (-1D/-1D) | Striae, high-arched palate | Connective tissue disorder | - | - | - | - | - | - | - | - | - |
| **28** | F | 8 | 2.2 | 0.4 | 1.7 | 1.2 | 2.2 | -0.3 | -0.5 | 0.1 | Myopia (-1.5/-2.0 D) | Joint hypermobility | Connective tissue disorder | - | - | - | - | - | - | - | - | - |
| **29** | F | 15 | 2.4 | -0.5 | 1.8 | 0.8 | 0.7 | 0.7 | 0.1 | -0.1 | AITD | 1 café au-lait spot, amblyopia, astigmatism, strabism | - | - | - | - | - | - | - | - | - | - |
| **30** | F | 11 | 2.8 | 0.4 | 0.7 | 0.6 | 0.7 | 0.6 | 0.1 | -1.6 | Hip dysplasia | Genua valga, strabism | - | - | - | - | - | - | - | - | - | - |
| **31** | M | 15 | 3.1 | 1.6 | 0.2 | 0.8 | 0.7 | 1.5 | 0.7 | -2.3 | - | 2 café au- lait spots | - | - | - | - | - | - | - | - | - | - |
| **32** | F | 10 | 2.0 | 0.3 | 0.7 | 0.5 | 0.8 | -0.1 | 0.1 | -3.2 | - | Positive wrist sign | - | - | - | - | - | - | - | - | - | - |
| **33** | M | 12 | 2.9 | 1.2 | 0.7 | 0.9 | 5.3 | 2.1 | 1.1 | -1.4 | Myopia (-2.5 D), astigmatism, | Joint hypermobility, | Connective tissue disorder | *TGFBR2* | Loyes – Dietz syndrome | NM_003242.6 | c.777T>A | p.Tyr259Ter | P | het | AD | - |

| | | | | | | | | | | | | | | | | | | | | | | |
|---|---|---|---|---|---|---|---|---|---|---|---|---|---|---|---|---|---|---|---|---|---|---|
| | | | | | | | | | | | retinoblastoma | skin hyperlaxicity | | | | | | | | | | |
| **34** | F | 16 | 2.7 | 1.7 | -0.04 | 0.8 | 1.1 | 1.1 | 0.5 | -1.2 | Myopia | High-arched palate | Connective tissue disorder | *COL1A2* | Ehlers Danlos syndrome arthrochalasia type 2, Osteogenesis imperfecta type 2,3,4 | NM_000089.4 | c.3978G>C | p.Lys1326Asn | VUS | het | AD, AR | - |
| **35** | F | 10 | 3.5 | 0.4 | 0.4 | 0.4 | 3.4 | 1.7 | 1.0 | -0.9 | Precocious puberty | Mild macrosomia | - | - | - | - | - | - | - | - | - | - |
| **36** | F | 7 | 2.3 | 0.8 | 0.7 | 0.8 | 0.2 | 0.3 | 0.1 | -1.3 | Spherocytosis, hypotonia | Macrocephaly | - | - | - | - | - | - | - | - | - | - |
| **37** | M | 15 | 2.8 | 1.2 | 0.2 | 0.7 | 0.7 | 1.2 | 1.1 | NA | ADHD | Macrosomia | - | - | - | - | - | - | - | - | - | - |
| **38** | F | 13 | 3.4 | -0.4 | 0.4 | 0.04 | 2.8 | 1.1 | 0.5 | -2.1 | AITD | Striae, genua valga | Connective tissue disorder | *COL6A3* | Bethlem myopathy | NM_004369.4 | c.3008G>A | p.Gly1003Glu | VUS | het | AD, AR | Pa |
| **39** | M | 16 | 2.9 | -0.4 | 1.4 | 0.5 | 0.1 | NA | NA | -1.4 | - | Striae, scoliosis, positive thumb sign | Connective tissue disorder | 47,XXY | Klinefelter syndrome | - | - | - | P | - | - | - |
| **40** | M | 19 | 2.4 | 1.4 | -0.04 | 0.6 | 1.1 | 1.5 | 0.7 | -1.8 | Burkitt lymphoma, myopia | Striae | - | - | - | - | - | - | - | - | - | - |
| **41** | M | 14 | 2.5 | -0.04 | 1.7 | 0.8 | 0.4 | 1.5 | 0.7 | 3.6 | Myopia (-0.8D/-0.8 D) | Striae | - | - | - | - | - | - | - | - | - | - |
| **42** | F | 11 | 3.7 | -0.04 | 1.5 | 0.8 | 1.5 | 0.9 | -0.4 | NA | - | Striae, scoliosis, genua valga | - | - | - | - | - | - | - | - | - | - |
| **43** | M | 10 | 2.5 | 1.7 | 0.2 | 0.9 | 0.4 | 2.0 | -0.2 | 0.4 | - | Joint hypermobility, skin hyperlaxity | Connective tissue disorder | *COL11A1* | Marshall-Stickler syndrome | NM_001854.4 | c.1351-3T>A | p.? | VUS | het | AD | - |
| **Children without dysmorphic features** | | | | | | | | | | | | | | | | | | | | | | |
| **44** | F | 9 | 2.4 | 1.4 | 1.4 | 1.5 | 3.4 | -2.4 | -2.2 | -1.6 | Precocious puberty | - | - | - | - | - | - | - | - | - | - | - |
| **45** | F | 6 | 3 | -0.5 | -0.04 | -0.3 | 2.0 | -0.1 | -0.8 | -0.5 | Granuloma anulare | - | - | - | - | - | - | - | - | - | - | - |

| | | | | | | | | | | | | | | | | | | | | | | |
|---|---|---|---|---|---|---|---|---|---|---|---|---|---|---|---|---|---|---|---|---|---|---|
| **46** | M | 15 | 2.7 | 0.3 | -0.6 | -0.2 | 0.7 | -0.6 | -0.7 | -1.9 | Obesity | - | - | - | - | - | - | - | - | - | - | - |
| **47** | F | 12 | 3.0 | 1.3 | 0.2 | 0.8 | -0.02 | 1.7 | 1.4 | -1.7 | AITD | - | - | - | - | - | - | - | - | - | - | - |
| **48** | F | 11 | 2.8 | 0.4 | 0.7 | 0.6 | 2.6 | 0.1 | 0.1 | 0 | - | - | - | *ZBTB2* | - | NM_001348800.3 | c.776G>A | p.Arg259His | VUS | het | - | Pa |
| **49** | M | 19 | 2.2 | 1.7 | 1.5 | 1.5 | -1.5 | 0.1 | -1.1 | 0 | Obesity, hypertension | - | - | - | - | - | - | - | - | - | - | - |
| **50** | M | 14 | 3.4 | -0.2 | -0.3 | -0.3 | -0.6 | 1.4 | 0.2 | -2.0 | Obesity, liver steatosis | - | - | *TET3* | Beck-Fahrner syndrome | NM_001287491.2 | c.3791A>G | p.Tyr1264Cys | VUS | het | AD, AR | Ma |
| **51** | M | 13 | 2.7 | -1.2 | 0.5 | -0.3 | 5 | 3.1 | 1.1 | -1.8 | Precocious puberty, astigmatism | - | - | - | - | - | - | - | - | - | - | - |
| **52** | F | 15 | 2.6 | 1.4 | 1.1 | 1.3 | 0.6 | -0.2 | 0.1 | -1.8 | - | - | - | - | - | - | - | - | - | - | - | - |
| **53** | F | 9 | 4.5 | 1.2 | 1.0 | 1.2 | 1.6 | 0.3 | 0.5 | -2.7 | - | - | - | - | - | - | - | - | - | - | - | - |
| **54** | F | 13 | 3.9 | 0.8 | 0.8 | 0.8 | 0.8 | 2.0 | 1.0 | -1.2 | - | - | - | - | - | - | - | - | - | - | - | - |
| **55** | F | 14 | 2.1 | -0.2 | 0.7 | 0.3 | 1 | 0.9 | -0.8 | -0.8 | Precocious puberty | - | - | - | - | - | - | - | - | - | - | - |
